# The digitalization and public crisis responses of small and medium enterprises: Implications from a COVID-19 survey

**DOI:** 10.1186/s11782-020-00087-1

**Published:** 2020-09-15

**Authors:** Hai Guo, Zhuen Yang, Ran Huang, Anqi Guo

**Affiliations:** 1 Business School, Renmin University of China, Beijing, 100872 China; 2 SME Development Research Center, Renmin University of China, Beijing, 100872 China

**Keywords:** COVID-19, Digitalization, Dynamic capabilities, Public crisis, Small and medium enterprises (SMEs)

## Abstract

The COVID-19 outbreak is a global crisis that has placed small and medium enterprises (SMEs) under huge pressure to survive, requiring them to respond effectively to the crisis. SMEs have adopted various digital technologies to cope with this crisis. Using a data set from a survey with 518 Chinese SMEs, the study examines the relationship between SMEs’ digitalization and their public crisis responses. The empirical results show that digitalization has enabled SMEs to respond effectively to the public crisis by making use of their dynamic capabilities. In addition, digitalization can help improve SMEs’ performance. We propose a theoretical framework of digitalization and crisis responses for SMEs and present three avenues for future research.

## Introduction

At the end of 2019, a novel coronavirus disease (COVID-19) broke out suddenly and spread rapidly to become a global pandemic. By late June 2020, COVID-19 had infected more than 8 million people worldwide, including more than 80,000 people in China. This public health crisis has posed great challenges for the survival and development of firms, with small- and medium-sized enterprises (SMEs) suffering in particular. The COVID-19 pandemic has been economically destructive in many ways. First, as more and more governments block cities in order to control the pandemic, the global supply chain has been significantly disrupted as both imports and exports are blocked. Second, delays in the resumption of work have greatly reduced firms’ production capacity while fixed costs such as salary and rent remain unchanged, leading to serious cash flow issues. Third, the reduction in demand due to the outbreak has posed serious threats to service sectors such as catering, hospitality, and cultural tourism. Worse, the damage caused by the COVID-19 outbreak is expected to be long-lasting and have a chilling effect on global economic growth.

SMEs play a vital role in promoting technological innovation, improving employment, and maintaining social stability (O’Regan et al. 2006). However, due to their shortage of resources, SMEs are much more vulnerable to public crises than other enterprises (Barron et al. 2012; Mayr et al. 2016). The existing literature has examined the roles of production recovery, corporate social responsibility, and community participation in reducing the threat of public crises on SMEs (Ballesteros et al. 2017; Kearins 2017; Neise and Diez 2019). In particular, firms’ dynamic capabilities have been found to be the key to public crisis responses (Lin and Wu 2014; Linnenluecke 2017; Martinelli et al. 2018). Unfortunately, the ways in which SMEs should build and leverage dynamic capabilities in public crises like the COVID-19 outbreak remain largely unclear.

In this study, we argue that digitalization has the potential to help SMEs respond effectively to public crises by activating their dynamic capabilities (Vial 2019). Digitalization refers to the use of digital technologies such as information, computing, communication, and connection technologies to promote organizational changes (Bharadwaj et al. 2013; Sebastian et al. 2017; Vial 2019). In the context of the COVID-19 outbreak, much research has suggested that the adoption of digital technologies plays an important role in crisis responses. In China, the government has encouraged the use of big data, artificial intelligence (AI), cloud computing, and other digital technologies in pandemic monitoring, virus tracing, disease treatment and work resumption. For example, big data technology can provide powerful support for real-time pandemic monitoring and tracking. The adoption of online office software enables employees to work remotely in a flexible manner.

Based on data from an online questionnaire survey conducted with 518 Chinese SMEs, the present study explores the relationships among digitalization, crisis response strategies to the COVID-19 outbreak, and the crisis response performance of SMEs. The survey results clearly show that digitalization can help SMEs employ emergency responses as well as respond strategically to public crises in the long run, thus contributing to the improvement in SMEs’ performance. Drawing on the dynamic capabilities perspective, we propose a theoretical framework of digitalization and public crisis responses and present several avenues for future research.

## Literature review

### Public crisis responses

Public crises are unexpected and disruptive external events that require organizations to make critical decisions under high pressure (Cui et al. 2016; Donaldson 1991; Smart and Vertinsky 1984). Coronavirus outbreaks such as those of SARS and COVID-19 are typical public crises (Pereira et al. 2019). First, the crisis outbreak is unexpected (Bundy et al. 2016), requiring firms to respond quickly. Second, the crisis is of high uncertainty (Lim et al. 2009; Pearson and Clair 1998), making it difficult to predict its impact. Third, the impact of the crisis is widespread (Burton et al. 1993). In the case of the COVID-19 pandemic, most industries across the world have suffered from it. The nature of public crises may lead to destructive consequences (Bundy et al. 2016; Martinelli et al. 2018; Noy 2009), by invalidating normal practices and rules and leading to huge economic losses and even humanitarian disasters (Wasileski et al. 2011).

Firms can be seriously affected by public crises. Such crises lead to instability, forcing firms to adjust their internal resources and capabilities to adapt to or operate upon the changing environment (Martinelli et al. 2018). As firms must respond to public crises as quickly as possible (Bundy and Pfarrer 2015; Helmer and Hilhorst 2006; Williams 2012), it is highly valuable to examine how firms should respond to such crises (Cui et al. 2016; Geroski et al. 2010; Mayr et al. 2016; Thornhill and Amit 2003).

Public crisis responses involve the posture that is taken with respect to modifying or adapting one’s actions in a turbulent crisis environment (Smart and Vertinsky 1984), which can be divided into two types: emergency responses in the short term for survival, and strategic responses in the long term for development (Müller 1985; Smart and Vertinsky 1984). In the short term, firms must make emergency responses to mitigate the immediate negative effects of a crisis (Müller 1985). Firms’ normal production and operation activities will be temporarily interrupted during the crisis (Martinelli et al. 2018), requiring them to implement immediate actions that promise immediate results, such as resuming production, improving efficiency, and reducing costs (Müller 1985). In addition, firms may have important social responsibilities in crisis areas (Neise and Diez 2019). They can obtain support from the government and the community to perform better during the crisis by undertaking social responsibilities (Kearins 2017), such as protecting the basic rights of employees and donating to communities (Ballesteros et al. 2017).

However, it is not sufficient to address ongoing threats (Müller 1985). In the long term, firms should strive to turn threats into opportunities, which requires strategic responses (Ginsberg 1988; Müller 1985; Smart and Vertinsky 1984; Smith and Sipika 1993). For example, the practical value of digitalization has been widely recognized in the context of the COVID-19 outbreak. Further, the crisis has also triggered strategic changes, including changes in product lines, market width, and external relations (Boeker 1997; Kirtley and O’Mahony 2020; Romanelli and Tushman 1994). Therefore, in the long term, firms should endeavor to pursue opportunities in the crisis environment through strategic responses (Wan and Yiu 2009; Wenzel et al. 2020).

The key to public crisis responses is to understand the crisis environment in a timely manner, seize opportunities, and reconfigure resources to cope with the crisis (Ballesteros et al. 2017; Yang and Hsieh 2013). Therefore, the dynamic capabilities perspective is highly relevant to crisis response research (Fainshmidt et al. 2017; Helfat 1997; Linnenluecke 2017; Martinelli et al. 2018). As the COVID-19 outbreak has been both unexpected and unpredictable, firms must possess dynamic capabilities to cope with it in a non-procedural, innovative, and dynamic manner.

### Dynamic capabilities and public crisis responses

Dynamic capabilities are the comprehensive capabilities to build, integrate, and reconfigure internal and external resources when coping with a rapidly changing environment (Teece 2007, 2012; Teece et al. 1997), which are regarded as powerful tools for firms to create and sustain value in a changing environment (Eisenhardt and Jeffrey 2000; Lin and Wu 2014). The turbulent nature of the environment becomes even more prominent in public crises. Thus, crises represent a real opportunity for firms to unleash the full potential of their dynamic capabilities (Linnenluecke 2017; Martinelli et al. 2018; Yang and Hsieh 2013).

In the context of a crisis, dynamic capabilities can be divided into three dimensions: the capability of *sensing* the crisis, the capability of *seizing* new opportunities in the crisis (Ballesteros et al. 2017; Teece 2007), and the capability of *reconfiguring* resources to cope with the crisis. First, firms with dynamic capabilities have the potential to sense or understand the crisis in a timely manner (Ballesteros et al. 2017; Lampel et al. 2009; Teece 2007). Admittedly, no firm could predict the arrival of the COVID-19 outbreak, but some firms may have sensed the spread of the outbreak and predicted that it would have a significant impact on their business. By assessing how the daily operations of the local community would be influenced by the potential crisis, such as the disruption of production and distribution activities, market failures, and staff shortages, firms can better perceive the crisis. Without sensing and understanding a crisis, it is virtually impossible to develop the comprehensive and interlinked strategies that are required to respond to it (Müller 1985).

Second, firms with dynamic capabilities are more likely to identify and capture new opportunities in a crisis (Ballesteros et al. 2017; Danneels 2002; Easterby-Smith et al. 2009). A public crisis breaks social patterns and gives birth to new business opportunities. For example, the shutdowns caused by the pandemic have provided opportunities for online businesses to flourish. Firms equipped with dynamic capabilities can better address valuable opportunities and are more likely to create and absorb new knowledge from the external environment, providing an impetus for changing under the crisis (Ballesteros et al. 2017; Makkonen et al. 2014). For instance, during the COVID-19 outbreak, Meituan launched the “unmanned delivery” plan, which involved contact-free deliveries, by restructuring its intelligent distribution system.

Third, firms can integrate and reconfigure their internal and external resources to cope with a crisis (Makkonen et al. 2014). Organizational inertia may pull firms away from observing external environment changes and adapting to them (Newey and Zahra 2009). As a result, firms without the capability to reconfigure their resources might fail to implement either short- or long-term crisis response strategies.

Overall, dynamic capabilities are critical for firms’ public crisis responses. Then comes the question: What kind of firms are more likely to be able to dynamically respond to public crises? To answer this question, this study highlights the important role of firms’ digitalization efforts, particularly under the context of the COVID-19 outbreak.

### Digitalization: a dynamic capabilities perspective

Digitalization refers to the process of organizational transformation through the adoption of digital technologies (Sebastian et al. 2017; Vial 2019), mainly manifested in organizations as digital artifacts, digital platforms, digital infrastructures (Briel et al. 2018; Giones and Brem 2017; Nambisan 2017; Nambisan et al. 2019; Yi et al. 2019), as well as digital business and management models (Srinivasan and Venkatraman 2018). Digital technologies are a mix of computerized information and communication technologies (Sturgeon 2019) and can be classified into seven types: social, mobile, big data, cloud computing, Internet of Things (IoT), platform development, and AI-related technologies (Sebastian et al. 2017; Vial 2019).

Digital technologies are programmable, addressable, senseable, communicable, memorable, traceable, and associable (Yoo 2010). Thus, digitalization or digital transformation can help firms gain and sustain competitive advantages by improving their organizational flexibility and resilience (Briel et al. 2018) and by enhancing their dynamic capabilities (Sambamurthy et al. 2003; Vial 2019). In particular, we propose that digitalization is beneficial to firms’ dynamic capabilities. First, digitalization helps firms sense environmental changes (Vial 2019; Warner and Maximilian 2018; Yoo 2010). The great advantage of digital resources in volume, velocity, variety, and value makes it possible for firms to collect or retrieve information resources in the external environment at a low cost (Gandomi and Haider 2015). In addition, the application of big data analysis systems and IoT technologies helps firms screen valuable information through high-speed computing so that they can sense and predict environmental changes to some extent (George et al. 2014; Sambamurthy et al. 2003).

Second, firms can better seize opportunities in a crisis environment with the help of digital technologies. In the COVID-19 outbreak, digitalization has created plentiful new opportunities (Nambisan et al. 2019), and areas such as online education, online working, and unmanned delivery have shown great potential. Further, the decentralized nature of digital technologies breaks obstacles in time and space, and promotes interactions between focal firms and their value co-creators, thus increasing their opportunities in open networks (Zeng and Glaister 2018). In addition, high-volume big data technology and high-speed cloud analysis technology have greatly improved the accuracy of business analysis, helping firms identify potential opportunities in complex environments (Briel et al. 2018). Furthermore, digital technology has changed the ways in which new opportunities are exploited, in ways that are more novel than predefined (Nambisan et al. 2019).

Finally, digitalization enables firms to reconfigure their resources to respond to crises. Digitalization improves firms’ available resources in scope, scale, and flexibility. For example, IT technologies reduce the cost of coordinating activities within firms and promote the flexible allocation of resources (Kane et al. 2015). In addition, digital technologies have fundamentally reshaped business processes, products, and services, as well as inter-firm relationships, greatly reducing the difficulty and costs of resource shifting (Nambisan et al. 2019). For instance, the blockchain, cloud computing, and IoT technologies have shortened the time required to launch new products and transform businesses, thus enabling firms to quickly adjust their operations with low costs (Warner and Maximilian 2018). During the COVID-19 outbreak, firms with a high degree of digitalization, such as Freshhema and Meituan, have been able to quickly reshape their businesses to minimize the adverse impacts or even benefit from the crisis.

## Survey methodology

### Sample and data collection

An online questionnaire survey was conducted to collect first-hand data. After reviewing relevant scales in the literature, the survey team discussed scale development and designed the questionnaire. In mid- to late-February 2020, the questionnaire was sent to firm managers through WeChat and other online social media channels. The respondents were mainly EMBA, MBA, and EE (executive education) students from key universities in China.

By February 23, 935 valid samples had been collected from 518 SMEs (with less than 500 employees), accounting for 55.4% of the full sample. As Table 1 shows, 66.02% of the sampled SMEs were private. Foreign-invested firms and state-owned firms account for 16.61% and 16.02% of the sampled SMEs, respectively. In terms of region, more than 80% of the SMEs were based in eastern China (including Shanghai, Jiangsu, Zhejiang, Fujian, Shandong, Jiangxi, and Anhui) and northern China (including Beijing, Tianjin, Hebei, Shanxi, and Inner Mongolia). The ratio of listed companies is relatively low, accounting for 10.42% of the sample. In addition, about 80% of the sampled firms’ business is conducted offline, leaving much room for digital transformation. The sampled firms are distributed across a wide range of industries, with manufacturing (26.64%); information transmission, software, and information technology (14.09%); and whole sales and retail trade (11.20%) ranking in the top three industries.

**Table 1:** Sample description

Business mode	Online	19.11	Industry	Agriculture, forestry, animal husbandry, and fishery	2.51
	Offline	80.89		Mining	0.97
Firm age	Under 6 years	12.93		Manufacturing	26.64
	6–10 years	13.51		Production and supply of electricity, heat, gas, and water	1.93
	11–15 years	15.83		Construction	3.28
	16–20 years	16.80		Transportation, warehousing, and postal	2.12
	More than 20 years	40.93		Information transmission, software, and information technology	14.09
Firm ownership	Private	66.02		Whole sales and retail trade	11.20
	Foreign	16.61		Catering and accommodation	1.74
	State	16.02		Finance	10.04
	Collective	1.35		Realty	2.90
Region	Northeastern China	0.59		Leasing and business services	4.25
	Northern China	39.01		Scientific research and technical services	4.44
	Central China	8.88		Water, environment, and utilities management	0.58
	Eastern China	41.12		Residential services, repairs, and other services	2.32
	Southern China	6.18		Education	3.67
	Western China	4.22		Health and social work	1.74
Listed	Listed	10.42		Culture, sports, and entertainment	4.05
	Unlisted	89.58		Public administration, social security, and social organization	0.58
Industry	Tourism	0.76		International organization	0.19

### Measurement

#### Digitalization

Three alternative measures were adopted to measure the digitalization degree of the sampled firms. First, digitalization can be defined as the *overall digitalization* degree of a firm, which is reflected through five items. (1) Digital artifacts refers to applications or media content with specific functions and values embedded in digital products or services, such as positioning applications in mobile phones to track travel trajectories during the pandemic (Ekbia 2009; Nambisan 2017). (2) A digital platform is a set of shared general services and architectures that plays as an important role as a carrier of digital artifacts (Nambisan 2017; Tiwana et al. 2010). (3) The digital infrastructure refers to digital technology tools and systems (Nambisan 2017). (4) The digital business model embodies the firm’s digital technology-driven value creation logic, such as online retail business during the COVID-19 outbreak. (5) The digital management model involves the application of digital technologies in the organizational management system, such as the adoption of intelligent office systems.

Second, we measure a firm’s digitalization through its *adoption of digital technologies*, which is reflected through seven items. These are (1) social, (2) mobile, (3) big data, (4) cloud computing, (5) IoT, (6) platform development, and (7) AI (Bharadwaj et al. 2013; Sebastian et al. 2017; Vial 2019).

Third, the digitalization degrees of firms for which the *businesses mode* is online are thought to be higher. Therefore, digitalization can be roughly measured by distinguishing online business from offline business (Biswas and Burman 2009).

#### Crisis response strategies

Firms’ public crisis response strategies can be classified into short-term emergency strategies and long-term strategic ones (Müller 1985; Smart and Vertinsky 1984). While short-term response strategies aim to adapt to the turbulent crisis environment, long-term oriented response strategies endeavor to identify opportunities for future development, suggesting the importance of dynamic capabilities (Ginsberg 1988; Müller 1985; Smart and Vertinsky 1984).

In this study, we consider three types of short-term response strategies: *production recovery strategies*, *employee protection strategies*, and *firm donation strategies* (Ballesteros et al. 2017; Neise and Diez 2019; Wenzel et al. 2020). Production recovery strategies are reflected through eight items: (1) reducing production and operating costs, (2) divesting loss-making/less-profitable business units, (3) adopting online telecommuting, (4) optimizing business models to capture new customer needs, (5) developing marketing channels and removing dependence on offline transactions, (6) actively investing in technological innovation, (7) diversifying into new business areas, and (8) integrating supply chain. Firms’ employee protection strategies are reflected through six items: (1) paying wages in accordance with contracts in one pay cycle, (2) paying basic subsistence allowance in excess of one pay cycle, (3) retaining employees’ jobs, (4) negotiating with employees or unions to defer payment, (5) paying wages to employees who are quarantined, and (6) arranging compensatory leave or overtime paying for employees who cannot take time off. The firms’ donation strategies are reflected through their donation amounts.

Further, two long-term oriented response strategies are included, namely *digital transformation* and *strategic changes* (Boeker 1997; Kirtley and O’Mahony 2020; Romanelli and Tushman 1994; Wan and Yiu 2009; Wenzel et al. 2020). Digital transformation is reflected through five items: (1) strengthening the application of online office tasks, (2) improving the digitalization of supply chain channels, (3) adopting digital artifacts, such as digital products or services, (4) adopting digital platforms, such as digital communication platforms, and (5) adopting digital infrastructures, such as digital technology systems. Strategic changes are reflected through three items: (1) changing existing product lines, (2) changing regional market coverage, and (3) changing external cooperative relations (Wan and Yiu 2009; Wenzel et al. 2020).

#### Crisis response performance

Four alternative measures were adopted to measure firms’ performance during the outbreak: *cost control status* reflected in the extent to which costs are controlled, *cash flow status* reflected in maintenance of cash flow over time, *revenue status in the first quarter* of 2020 reflected in the extent to which revenue declines are controlled, and overall *predicted performance* under the crisis reflected in the perception of the firm’s overall performance.

Unless specifically explained, the scales adopted in this study are all five-point Likert scales, with 1 indicating “totally disagree,” “almost none,” or “very poor” and 5 indicating “totally agree,” “pretty much,” or “very good.” We measured the value of each variable by taking the average value of all measuring items. To make the statistical results more intuitive, the average value interval of most variables was converted into an interval from 0 to 1, except for the minimum and maximum.

## Survey results

### Descriptive and correlational analysis for key variables

Table 2 shows the descriptive statistics for key variables used in this study. First, in terms of the degree of digitalization, the results show that Chinese SMEs have made initial achievements in digitalization, but there is still plenty of room for improvement. Specifically, the mean of digital artifacts (usually embedded in products and services), digital platforms, and digital infrastructure are all above 0.6, indicating that Chinese SMEs have made initial attempts at digitalizing. However, the digital ability in value creation is insufficient, as the business model (0.56) and management model (0.58) are less digitalized. Second, our results show that compared to internal R&D, SMEs often resort to external technologies for digitalization. Third, in terms of digital technology adoption, the values for social technology (0.69) and mobile technology (0.68) are higher. This can be credited to the rapid development of China’s e-business and Internet economy over the past 20 years. However, the adoption of the latest digital technologies such as AI (0.59) and cloud computing (0.62) still requires improvement. Finally, considering the modes of business, the online rate is insufficient (0.19).

**Table 2:** Descriptive statistics for key variables

	Dimension	Observation	Mean	S.D.	Min	Max
Overall digitalization degree	Digital artifact	518	0.61	0.22	1	5
	Digital platform	518	0.63	0.22	1	5
	Digital infrastructure	518	0.60	0.21	1	5
	Digital business model	518	0.56	0.22	1	5
	Digital management model	518	0.58	0.21	1	5
Digitalization method	Internal R&D	518	0.55	0.25	1	5
	External acquisition	518	0.61	0.22	1	5
Digital technology adoption	Big data	518	0.62	0.23	1	5
	AI	518	0.59	0.24	1	5
	Mobile	518	0.68	0.22	1	5
	Cloud computing	518	0.62	0.23	1	5
	IoT	518	0.59	0.24	1	5
	Social	518	0.69	0.22	1	5
	Platform development	518	0.61	0.23	1	5
Business mode	Rate of online business	518	0.19	0.39	0	1
Short-term crisis responses	Production recovery	518	0.65	0.16	1	5
	Employee protection	518	0.71	0.15	1	5
	Donation	518	0.32	0.17	1	5
Long-term crisis responses	Digital transformation	518	0.71	0.21	1	5
	Strategic change	518	0.54	0.20	1	5
Performance	Cost control status	518	0.70	0.25	1	6
	Cash flow status	518	0.59	0.26	1	5
	Revenue status in the first quarter	518	0.56	0.24	1	6
	Predicted performance	518	0.59	0.20	1	5

In terms of short-term crisis responses, SMEs have generally adopted production recovery (0.65) and employee protection strategies (0.71), while the adoption rate of the donation strategy is relatively low (0.32). These results suggest that the most important task for SMEs is to survive the crisis. For long-term crisis responses, SMEs prefer to implement digital transformation strategies (0.71) instead of strategic changes in products, markets, and external relations (0.54). These results imply that SMEs have realized the unique value of digitalization in the COVID-19 outbreak.

Regarding crisis response performance, the results of the survey show that SMEs have been adversely affected by the COVID-19 pandemic in various aspects. Costs (0.70) have increased less than 10%, but revenue in the first quarter (0.56) has decreased by 10%–50% on average, and cash flow (0.59) can only be maintained for about half a year, on average. Overall, under the COVID-19 outbreak, there is an inevitable decline in predicted performance (0.59) compared with firms’ performance in 2019.

Table 3 shows descriptive statistics with correlations of variables. *Province* (the province in which the firm is located), *industry*, *property*, and *year* (the year in which the firm was established) are set as control variables. The results show that the digitalization of SMEs is positively associated with the implementation of crisis response strategies and performance, and that crisis response strategies are also positively associated with performance. Therefore, it is necessary to continue to explore the functional relationship between variables. We calculated the variance inflation factor (VIF) and the result shows that the maximum value is 1.10, far below the cutoff value of 10. Our results show no evidence of multicollinearity among all variables.

**Table 3:** Results of correlations analysis

	(1)	(2)	(3)	(4)	(5)	(6)	(7)	(8)	(9)	(10)
(1) Province	1.000									
(2) Industry	−0.006	1.000								
(3) Property	0.138**	0.266***	1.000							
(4) Year	0.024	−0.017	−0.011	1.000						
(5) Overall degree of digitalization	− 0.069	0.047	− 0.037	0.009	1.000					
(6) Digital technology adoption	−0.075	0.049	0.007	−0.066	0.702***	1.000				
(7) Business mode (online/offline)	−0.110**	0.057	−0.002	−0.023	0.247***	0.249***	1.000			
(8) Short-term crisis responses	0.021	−0.045	− 0.070	−0.068	0.287***	0.352***	0.046	1.000		
(9) Long-term crisis responses	0.014	−0.031	− 0.045	−0.120**	0.296***	0.383***	0.079*	0.464***	1.000	
(10) Performance	−0.006	−0.030	− 0.131**	−0.042	0.109**	0.115	0.052**	0.154***	−0.001	1.000

* *p* < 0.05; ** *p* < 0.01; *** *p* < 0.001

### Digitalization and responses to the COVID-19 outbreak

As this is a survey study, a more intuitive and concise approach should be adopted to clearly present the results in a simple manner. To this end, we divided the value of each variable into three degrees, namely low, medium, and high. For example, the value of the degree of digitalization was sorted from the largest to the smallest, and then divided into three equal groups according to the number of samples, namely the high group, the middle group, and the low group. We then calculated the average value of the variable at each degree. This grouping method has the advantages of simplicity, intuition, and clarity in the presentation of data, which enhances the readability of the paper. The values were reserved to two decimal places, except for special notes.

As shown in Table 4, the survey results generally indicate that the digitalization of SMEs is positively associated with the implementation of crisis response strategies, including both short-term emergency responses (*p* < 0.05) and long-term strategic responses (*p* < 0.001). In the face of the COVID-19 outbreak, highly digitalized SMEs can more effectively use short-term responses. For SMEs with a high degree of digitalization or digital technology adoption, the average scores of short-term crisis responses are 0.62 and 0.64, respectively, both of which are higher than those for SMEs with a medium or low degree of digitalization. Specifically, in terms of digitalization, the scores for production recovery strategy, employee protection strategy, and donation strategy are 0.70, 0.74, and 0.34, respectively. In terms of digital technology adoption, the three scores are 0.71, 0.75, and 0.34, respectively. The policy-based shutdown under the pandemic has left many firms facing production stagnation. Highly digitalized firms with dynamic capabilities are more likely to integrate their internal and external resources quickly to resume production and operation activities through methods such as adopting online telecommuting or divesting less-profitable units. In addition, digital firms can make donations to the pandemic area through existing digital channels. Internet firms, which have attracted plenty of well-deserved attention during this pandemic, have higher philanthropic efficiency than traditional firms and charities.

**Table 4:** Digitalization and responses to the COVID-19 outbreak

	Short-term crisis responses			Long-term crisis responses		Overall response
	Production recovery	Employee protection	Donation	Digital transformation	Strategic change	Mean
Overall digitalization degree						
Low	0.56	0.67	0.28	0.57	0.46	0.51
Medium	0.64	0.71	0.31	0.70	0.55	0.58
High	0.70	0.74	0.34	0.76	0.58	0.62
*p*-value	0.000	0.001	0.007	0.000	0.000	0.000
Performance	+	+	+	+	+	+
Digital technology adoption						
Low	0.50	0.66	0.25	0.54	0.42	0.47
Medium	0.64	0.70	0.30	0.68	0.53	0.57
High	0.71	0.75	0.34	0.78	0.60	0.64
*p*-value	0.000	0.000	0.000	0.000	0.000	0.000
Performance	+	+	+	+	+	+
Business mode						
Online	0.69	0.71	0.33	0.72	0.58	0.61
Offline	0.65	0.71	0.31	0.54	0.54	0.55
*p*-value	0.236	0.948	0.270	0.196	0.073	0.067
Performance	#	#	#	#	Online better	Online better

“+”: The higher, the better; “-”: The lower, the better; “#”: No difference

Similarly, highly digitalized SMEs are more determined to make long-term responses in order to deal with the crisis. For SMEs with a high degree of digitalization, the average scores of digital transformation intention and strategic change intention are 0.76 and 0.58, respectively, all higher than those for SMEs with a medium or low degree of digitalization. Similarly, for SMEs with a high degree of digital technology adoption, the average scores of digital transformation intention and strategic change intention are 0.78 and 0.60, respectively, all higher than those for SMEs with a medium or low degree of digital technology adoption. The agility and openness of digital technologies greatly improves the accuracy of business analysis for the firms that employ such technologies. This explains why highly digitalized firms are more likely to find potential opportunities in disruptive environments and integrate resources for strategic transformation and changes.

In addition, SMEs, with the primary business conducted online instead of offline, will perform better in responding to the COVID-19 outbreak (0.61 vs. 0.55, *p* < 0.1), which is mainly reflected in their long-term crisis responses (0.58 vs. 0.54, *p* < 0.1). Specifically, regarding other crisis response strategies, the performance of SMEs that conduct their business online is no worse than that of those that conduct their business offline, and the difference is not highly significant.

### Responses to the COVID-19 outbreak and response performance

Table 5 shows the relationship between SMEs’ crisis response strategies and performance outcomes. Overall, both short- and long-term strategies in response to the COVID-19 outbreak will lead to improved performance for firms.

**Table 5:** Responses to COVID-19 outbreak and response performance

		Performance				Overall performance
Short-term crisis responses	Production recovery	Cost control	Cash flow	Revenue	Predicted performance	Mean
	Low	0.57	0.58	0.46	0.57	0.55 (0.545)
	Medium	0.58	0.60	0.46	0.58	0.56 (0.555)
	High	0.59	0.58	0.48	0.60	0.56 (0.563)
	*p*-value	0.586	0.325	0.386	0.099	0.325
	Performance	#	#	#	+	#
	Employee protection	Cost control	Cash flow	Revenue	Predicted performance	Mean
	Low	0.59	0.57	0.45	0.56	0.54 (0.542)
	Medium	0.55	0.63	0.48	0.59	0.56 (0.562)
	High	0.60	0.57	0.47	0.60	0.56 (0.560)
	*p*-value	0.969	0.257	0.193	0.021	0.104
	Performance	#	#	#	+	#
	Donation	Cost control	Cash flow	Revenue	Predicted performance	Mean
	Low	0.57	0.54	0.46	0.58	0.54
	Medium	0.59 (0.590)	0.60	0.45	0.56	0.55
	High	0.59 (0.591)	0.63	0.49	0.61	0.58
	*p*-value	0.317	0.000	0.005	0.005	0.000
	Performance	#	+	+	+	+
Long-term crisis responses	Digital transformation	Cost control	Cash flow	Revenue	Predicted performance	Mean
	Low	0.59	0.54	0.46	0.59	0.55
	Medium	0.57	0.59	0.47 (0.471)	0.58	0.55
	High	0.58	0.64	0.47 (0.473)	0.59	0.57
	*p*-value	0.876	0.004	0.422	0.448	0.088
	Performance	#	+	#	#	+
	Strategic change	Cost control	Cash flow	Revenue	Predicted performance	Mean
	Low	0.57	0.58	0.49	0.60	0.56
	Medium	0.58	0.60	0.47	0.59	0.56
	High	0.60	0.59	0.45	0.57	0.55
	*p*-value	0.028	0.859	0.020	0.128	0.026
	Performance	+	#	–	#	–

“+”: The higher, the better; “-”: The lower, the better; “#”: No difference

On the one hand, short-term crisis response strategies can improve the performance of SMEs, though this does not occur in a uniform manner. Production recovery strategies (0.60) and employee protection strategies (0.60) can lead to better predicted performance (*p* < 0.1), but do not necessarily help SMEs improve their current performance. Excessive production recovery and employee protection will consume a large amount of a firm’s resources, putting even greater pressure on already thinly stretched SMEs during the outbreak. Therefore, a moderate degree of production recovery and employee protection may be a better option. Donation strategies have an overall positive impact on SME performance (0.58) and can help SMEs obtain more revenue (0.49), leading to stronger cash flow (0.63) and better predicted performance (0.61). SMEs and stakeholders are a community of interests in the public crisis. By donating to relevant causes, SMEs can obtain support from the government and the community to mitigate the harm of the crisis in an atmosphere of mutual assistance.

On the other hand, long-term crisis response strategies have inconsistent effects on SMEs’ performance. Digital transformation strategies have the potential to generate better performance for SMEs, especially related to cash flow maintenance (0.64). However, strategic changes are associated with lower SME performance (0.55), indicating that an SME’s intention to change its strategy seems to merely be a reflection of poor performance. Strategic changes will consume a large amount of human, material, and financial resources, placing huge pressure on SMEs that are already struggling in the outbreak. However, this does not mean that strategic changes are not a good choice because the benefits of successful changes may be long-term and sustainable even if the temporal performance may decline.

### Digitalization and crisis response performance

As shown in Table 6, the results of the survey indicate that digitalization is positively associated with SMEs’ crisis response performance.

**Table 6:** Digitalization and crisis response performance

	Performance				Overall performance
	Cost control	Cash flow	Revenue	Predicted performance	Mean
Overall digitalization degree					
Low	0.59	0.50	0.45	0.56	0.53
Medium	0.57	0.57	0.45	0.57	0.54
High	0.60	0.66	0.50	0.61	0.59
*p*-value	0.895	0.003	0.096	0.046	0.016
Performance	#	+	+	+	#
Digital technology adoption					
Low	0.55	0.51	0.42	0.54	0.51
Medium	0.59	0.56	0.46	0.58	0.55
High	0.58	0.63	0.48	0.60	0.57
*p*-value	0.934	0.000	0.120	0.103	0.009
Performance	#	+	#	#	+
Business mode					
Online	0.59	0.58 (0.582)	0.49	0.59	0.56
Offline	0.58	0.58 (0.578)	0.46	0.58	0.54
*p*-value	0.688	0.329	0.243	0.457	0.235
Performance	#	#	#	#	#

“+”: The higher, the better; “-”: The lower, the better; “#”: No difference

First, a high degree of digitalization will lead to higher SME performance (0.59, *p* < 0.05). Specifically, digitalization could help SMEs maintain cash flow (0.66, *p* < 0.05), generate more revenue (0.50, *p* < 0.1), and lead to perceived best predicted performance (0.61, *p* < 0.05). As identified above, highly digitalized firms are more likely to resume production, effectively curbing revenue and profit declines. However, the advantage of digitalization for cost control is not obvious, leading to a lack of significant advantages for firms’ overall performance.

Second, similarly, a high degree of digital technology adoption is associated with optimal SME performance (0.57, *p* < 0.05). Specifically, the adoption of digital technologies is associated with a higher degree of cash flow maintenance (0.63, *p* < 0.001), all higher than the rest. However, this cannot help SMEs control costs or obtain revenue most effectively.

Finally, for SMEs that conduct their primary business online, their performance outcomes are not worse than those whose business is conducted offline, but the difference is not significant. The simple descriptive statistics show that compared to offline businesses, online ones may score consistently higher in cost control (0.59), cash flow maintenance (0.58), revenue (0.49), and predicated performance (0.59). However, these results require further and more rigorous verification.

Table 7 shows the ways in which the sampled SMEs have adopted different digital technologies to improve their crisis response performance. The results indicate big data, mobile, and cloud computing technologies are particularly effective in helping SMEs cope with the crisis, but this does not occur in a uniform manner. First, mobile technologies are particularly helpful for cost control (*β* = 0.100, *p* < 0.05). Technologies such like 4G, instant messaging, and GPS together with a variety of mobile applications could support SMEs in refined management and improve their organizational efficiency. For example, trajectory tracking applications can help SMEs efficiently conduct human resource management. Second, big data technologies are regarded as most useful for maintaining cash flow (*β* = 0. 221, *p* < 0.001) and generating revenue (*β* = 0. 098, *p* < 0.05). In the digital economy, big data not only helps firms improve the efficiency of their existing businesses, it also enables the creation of new businesses. During the COVID-19 pandemic, big data technology may not only help SMEs evaluate the spread of the pandemic, guiding them to reactivate work and production, it may also help them identify and take advantage of new business opportunities in the crisis and thus guide the direction of future developments. Finally, cloud computing technology seems to be most important for SMEs’ overall predicted performance (*β* = 0. 076, *p* < 0.05). This indicates that a new era of cloud computing is being welcomed.

**Table 7:** Digital technology adoption and crisis response performance

	Performance				Overall performance
Digital technology adoption	Cost control	Cash flow	Revenue	Predicted performance	Mean
Social	0.032	0.152**	0.079	0.074†	0.050†
Mobile	0.100*	0.148**	0.070	0.050	0.091**
Cloud computing	0.030	0.167***	0.081†	0.076*	0.089**
Big data	0.005	0.221***	0.098*	0.035	0.090**
IoT	−0.042	0.167***	0.047	0.043	0.046
Platform development	−0.052	0.152***	0.053	0.049	0.054
AI	−0.034	0.162***	0.007	0.040	0.044
Optimal technology	Mobile	Big data	Big data	Cloud computing	Big data & mobile & cloud computing

† *p* < 0.10; * *p* < 0.05; ** *p* < 0.01; *** *p* < 0.001

## Robustness test

Descriptive analyses and simple correlation analyses are often conducted in survey research. However, entirely abandoning the regression may cause problems hazarding research rigor such as neglecting the partial correlation variables. Therefore, controlling the four variables of *province*, *industry*, *property*, and *year*, we conducted a simple whole regression test among the main variables as a robustness test.

As shown in Table 8, first, the overall degree of digitalization has a significant positive impact on the implementation of crisis response strategies, including both short-term responses (*β* = 0.161, *p* < 0.001) and long-term responses (*β* = 0.265, *p* < 0.001). Second, digital technology adoption also has a significant positive impact on the implementation of short-term responses (*β* = 0.192, *p* < 0.001) and long-term crisis responses (*β* = 0.328, *p* < 0.001). Third, SMEs that conduct their primary business online instead of offline will perform better in crisis responses, especially in long-term responses (*β* = 0.177, *p* < 0.1). In addition, overall digitalization degree (*β* = 0.090, *p* < 0.01) and digital technology adoption (*β* = 0.095, *p* < 0.01) both have a significant positive impact on SMEs’ performance. The implementation of crisis response strategies is further positively associated with SMEs’ performance, which is mainly reflected in their short-term strategies (*β* = 0.218, *p* < 0.001). These empirical results support our arguments.

**Table 8:** Results of regression analysis

	Short-term crisis responses			Long-term crisis responses			Performance				
Province	0.239	0.163	0.401	0.322	0.212	0.472	0.650	0.620	0.668	0.841	0.758
Industry	0.303	0.296	0.496	0.375	0.356	0.571	0.991	0.994	0.953	0.830	0.906
Property	0.211	0.108	0.141	0.479	0.282	0.337	0.005**	0.003**	0.003**	0.006**	0.003**
Year	0.082*	0.248	0.115	0.003**	0.016*	0.006**	0.305	0.406	0.331	0.441	0.308
Overall digitalization degree	0.161***			0.265***			0.090**				
Digital technology adoption		0.192***			0.328***			0.095**			
Business mode (online/offline)			0.068			0.177†			0.109		
Short-term crisis responses										0.218***	
Long-term crisis responses											0.012
*F*-value	10.85***	16.24***	1.46	12.32***	19.84***	2.54**	3.21**	3.42**	2.30*	4.21***	2.02†
*R*^2^	0.096	0.137	0.014	0.107	0.162	0.024	0.030	0.032	0.022	0.040	0.019
Adjusted *R*^2^	0.087	0.129	0.004	0.099	0.154	0.015	0.021	0.023	0.012	0.030	0.010

* *p* < 0.05; ** *p* < 0.01; *** *p* < 0.001

## A summary of the theoretical framework

**Fig. 1:**
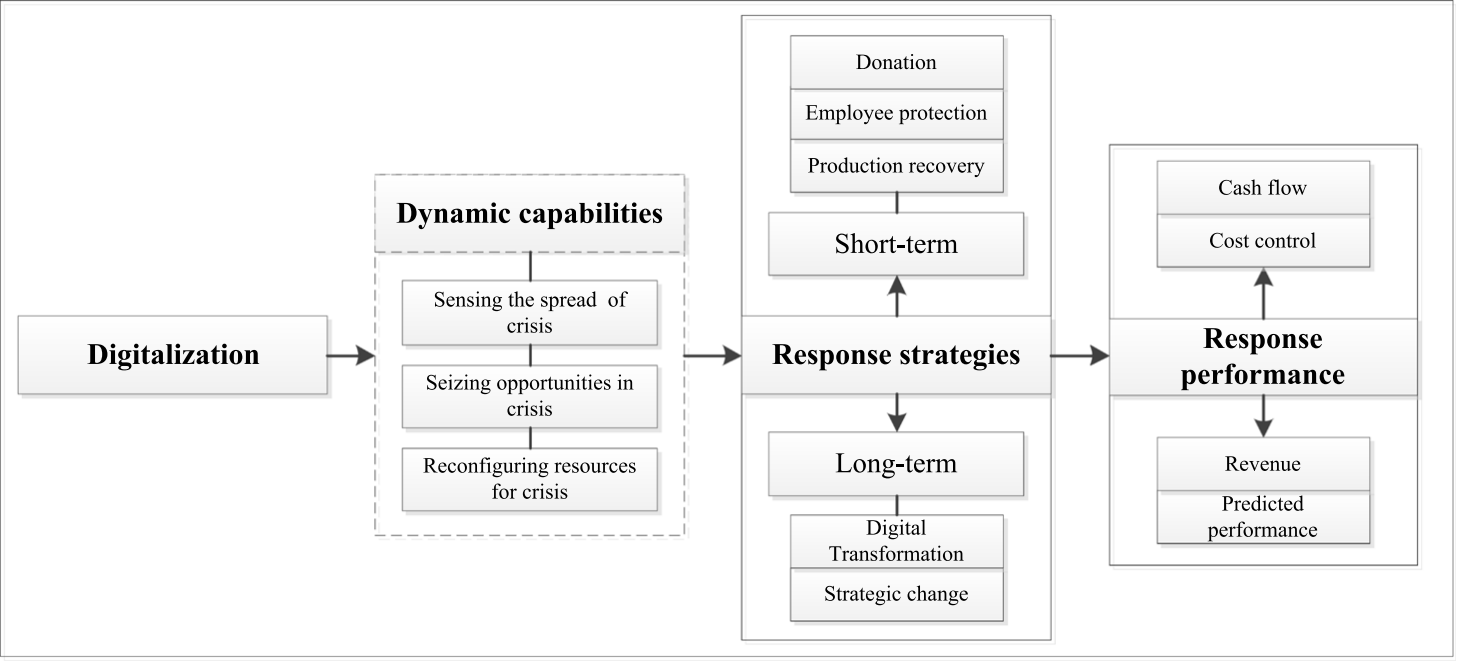
Digitalization and public crisis responses: A theoretical framework

We have constructed a theoretical framework based on the above survey results (Fig. 1). The survey results show that digitalization is positively associated with SMEs’ public crisis response strategies and performance, which indicates that SMEs with a higher degree of digitalization are more likely to adopt effective public crisis response strategies and achieve better performance during the COVID-19 outbreak. Drawing on the dynamic capabilities perspective, we emphasized the role digitalization activities play in firms’ crisis responses. Highly digitalized firms can leverage their dynamic capabilities to sense a crisis, seize opportunities during the crisis, and reconfigure resources to cope with the crisis (Vial 2019; Warner and Maximilian 2018; Yoo 2010), which means these firms are more likely to respond to crises quickly and effectively. Further, crisis response strategies, including short-term emergency responses and long-term strategic responses (Ballesteros et al. 2017; Smith and Sipika 1993), can lead to improved performance for SMEs. Here, we propose three avenues for future research.

### Topic 1: digitalization and dynamic capabilities

Digital technologies are at the center of digitalization. Sebastian et al. (2017) further builds an SMACIT^1^ framework to classify various digital technologies. The sensitivity and openness of digital technologies provide flexible access to and enable agile responses to the external environment. Existing literature shows that digitalization can significantly improve firms’ dynamic capabilities (Vial 2019; Warner and Maximilian 2018; Yoo 2010), enabling them to remain flexible in highly uncertain environments (Briel et al. 2018). Compared with traditional firms, digitalized firms are better prepared to sense the environment (George et al. 2014; Nylen and Holmstrom 2015; Vial 2019), to seize opportunities (Briel et al. 2018), and to reconfigure resources (Vial 2019).

However, the connotations of dynamic capabilities are changing under the context of digital transformation. On the one hand, the widespread use of digital technologies is reshaping the nature and purpose of dynamic capabilities (Warner and Maximilian 2018), as the powerful aggregation and generation of digital technologies elevate firms’ dynamic capabilities. For instance, blockchain, cloud computing, and IoT technologies enable firms to exponentially expand the scope and scale of their businesses. Therefore, digital dynamic capabilities may become a key source of competitive advantages for firms in the digital economy (Warner and Maximilian 2018). On the other hand, the boundary of dynamic capabilities is also changing. For example, Makkonen et al. (2014) reclassify dynamic capabilities into regenerative and renewing capabilities, which are manifested by indicators like reconfiguration, leveraging, learning, sensing and seizing, knowledge creation, and knowledge integration.

Thus, future research should determine how digitalization reshapes firms’ dynamic capabilities. Some research questions related to this topic are listed below. First, what is digitalization and what do digitalization activities involve? Academic terms such as digitalization, digital technology adoption, digital innovation, and digital transformation should be carefully defined. Second, how should a digital or digitalized firm be defined? Third, what are digital dynamic capabilities? In other words, what is the nature and boundary of dynamic capabilities in the digital economy? Fourth, how will the adoption and combination of multiple digital technologies impact firms’ dynamic capabilities? Finally, how should a firm construct its digital dynamic capabilities?

### Topic 2: digitalization and public crisis response strategy

Firms now operate in “VUCA” times, characterized by volatility, uncertainty, complexity, and ambiguity (Bennett and Lemoine 2014). We have witnessed more and more crises in recent years. In fact, at least four public crises have occurred since the start of 2020, including the COVID-19 outbreak, the African locust plague, the collapse of oil prices, and the U.S. stock market meltdown, all reminding us that we should pay attention to responses. Firms should build, integrate, and reconfigure resources to cope with complex, turbulent, and highly uncertain environments (Bennett and Lemoine 2014; Smart and Vertinsky 1984). The world is undergoing a new industrial revolution—the digital revolution (Rindfleisch et al. 2017). Digitalization creates an abundance of business opportunities, and it is worth questioning whether it may help firms survive or even benefit from crises.

However, the existing literature on firms’ crisis responses is focused on the firm level, such as bankruptcy, stock price decline, and reputation damage (Mayr et al. 2016; Snyder et al. 2006; Wei et al. 2017), and public crisis responses have been examined less frequently. Further, existing studies on public crises often address issues like governance and community resistance (Donaldson 1991; Martinelli et al. 2018), overlooking the role of firms. The findings of our survey clearly indicate that digitalization can be useful in public crisis responses. Compared with other firms, digitalized firms are more likely to adopt short-term and long-term crisis responses and enjoy better performance outcomes. Further, we have bridged digitalization and crisis responses from the perspective of dynamic capabilities. However, research on this theoretical bridge is still in its infancy, with many deficiencies. Dynamic capability theory is not the only theory that may help us understand digitalization and crisis responses. Other traditional management theories have the potential to be integrated with digitalization, and the theory of digitalization itself deserves more exploration.

Therefore, it is valuable to examine the relationship between digitalization and firm-level crisis response strategies. We suggest the following research questions. First, how do different public crises (natural vs. social) reshape the business environment? Second, what is the nature of firms’ strategic decision-making in crisis environments characterized by sudden and violent environmental changes? Third, what are the similarities and differences between short-term emergency response strategies and long-term strategic response strategies? Finally, what is the underlying mechanism through which digitalization impacts crisis response strategies?

### Topic 3: digitalization and firms’ competitive advantages

The results of the survey indicate that digitalization has a positive effect on SMEs’ performance. Although this paper only focuses on SMEs’ present performance, future research should consider both present and future performance, as digitalization strategies have long-lasting impacts on firms. Digitalization has greatly altered the ways in which businesses are run (Ofek and Wathieu 2010), which can be a new source of competitive advantages for firms. On the one hand, digitalization can improve a firm’s operational efficiency by automating decision-making, improving the efficiency of business processes, and saving costs (Andriole 2017; Pagani 2013). For example, cloud computing technology provides elastic resources, which reduces the cost of hiring, managing, and maintaining technology talents (Kane et al. 2015). Big data technology accelerates firms’ decision-making processes, increasing the speed at which they can respond to intelligent products and services (Bharadwaj et al. 2013). On the other hand, digitalization involves digital innovation activities (Li et al. 2017) such as creating new products, services, business models, and organizational forms (Autio et al. 2018; Yoo 2010).

As digitalization often occurs at the firm level, firms must make holistic strategic deployments (Fitzgerald 2014), that is, digital transformation strategies. However, it remains unclear how firms should design and implement their digital transformation strategies. Further, although the significance of digital transformations has been recognized, there is still a lack of a theoretical framework that can guide firms to realize this transformation. Vial (2019) asserts that digital transformation will contribute to firms’ performance through the design of digital business strategies, the adoption of digital technologies, and changes of value creation paths. However, it is unclear how this process works.

It is thus a promising research direction to explore digital transformation strategies as well as their impact on firms’ competitive advantages. We suggest the following research questions. First, is digitalization a new source of competitive advantages for firms? Second, how should firms’ digital business strategies be designed? Third, how does a digital business strategy guide a firm’s adoption of digital technologies? Finally, how does digital technology adoption change a firm’s value creation paths?

## Conclusion

The COVID-19 outbreak is a public health crisis that has posed great challenges for the survival and growth of SMEs. The crisis has also highlighted the important role of digital technologies in the response to the COVID-19 outbreak. Data from a questionnaire survey was used in this study to investigate the relationships among digitalization, public crisis responses, and SME performance in the context of the COVID-19 outbreak. The results of our survey show that an SME’s efforts towards digitalization, manifested by their degree of digitalization, adoption of digital technologies, and business mode can help them better respond to public crises. Further, digitalization contributes to improvements in SMEs’ performance through the implementation of public crisis response strategies. To conclude the study, we construct a theoretical framework that links digitalization with public crisis responses from the dynamic capabilities perspective and propose three avenues for future research.

## Data Availability

The datasets used or analyzed during the current study are available from the corresponding author on reasonable request.

## Appendix^2^

### Digitalization

**1. What is your firm’s overall degree of digitalization?**

**Table:**

	Totally disagree	Partially disagree	Not sure	Partially agree	Totally agree
We fully adopt digital artifacts (products or services)					
We fully adopt digital platforms that support digital products and services					
We fully adopt digital infrastructures, such as technology tools and systems					
We fully adopt digital business models					
We fully adopt digital management models					
*Firm digitalization relies on internal R&D*					
*Firm digitalization relies on external purchases*					

*Notes.* Items 6 and 7 are only used to distinguish firms’ digitalization orientations.

**2. What is your firm’s degree of digital technology adoption?**

**Table:**

	Very low	Low	Not sure	High	Very high
Big data technology (such as big database, data analysis technology)					
AI technology (such as machine learning)					
Mobile technology (such as mobile Internet, wireless communications)					
Cloud computing technology (such as cloud computing)					
IoT technology (such as network distribution technology)					
Social technology (such as online commerce, instant messaging)					
Platform development technology (such as network platforms)					

**3. Your firm’s business of is mainly:**

**Table:**

A. Online	B. Offline

### Public crisis response strategy

**1. In face of the pandemic, your firm has taken the following strategies to resume production:**

**Table:**

	Totally disagree	Partially disagree	Not sure	Partially agree	Totally agree
Reduce production and operating costs					
Divest loss-making/less profitable business units					
Adopt online telecommuting					
Optimize business models to capture new customer needs					
Develop marketing channels and remove dependence on offline transactions					
Actively invest in technological innovation					
Diversify into new business areas					
Integrate supply chain					

**2. In the face of the pandemic, your firm has taken the following strategies to protect employees’ rights:**

**Table:**

	Totally disagree	Partially disagree	Not sure	Partially agree	Totally agree
Pay wages in accordance with contracts in one pay cycle					
Pay basic subsistence allowance in excess of one pay cycle					
Retain employees’ jobs					
Negotiate with employees or unions to defer payment					
Pay wages to employees who are quarantined					
Arrange compensatory leave or overtime pay for employees who cannot take time off					

**3. Has your firm donated money or materials to the pandemic area so far? If so, what is the total amount?**

A. No donations or supplies have been made
B. Less than 1 million
C. 1 million–5 million
D. 5 million–10 million
E. More than 10 million

**4. Will your firm change in the following aspects after the pandemic?**

**Table:**

	Totally disagree	Partially disagree	Not sure	Partially agree	Totally agree
Change existing product lines					
Change regional market coverage					
Change external cooperative relations					

**5. Will your firm accelerate its digital transformation after the pandemic?**

**Table:**

	Totally disagree	Partially disagree	Not sure	Partially agree	Totally agree
Strengthen the application of online office tasks					
Improve the digitalization of supply chain channels					
Adopt digital artifacts, such as digital products or services					
Adopt digital platforms, such as digital communication platforms					
Adopt digital infrastructures, such as digital technology systems					

### Public crisis response performance

1. **Compared with its performance in 2019, how will your firm’s performance change in the future?**
  A. Decrease more than 50%
  B. Decrease by 30%–50%
  C. Decrease less than 30%
  D. No change
  E. Increase
2. **How long can you maintain the cash flow of your firm during the pandemic?**
  A. 1 month
  B. 3 months
  C. About half a year
  D. About 1 year
  E. More than 1 year
3. **How will your firm’s revenue in the first quarter change during the pandemic?**
  A. Decrease more than 90%
  B. Decrease by 50%–90%
  C. Decrease by 10%–50%
  D. Decrease less than 10%
  E. No change
  F. Increase
4. **How will the costs of your firm change during the pandemic?**
  A. Increase more than 100%
  B. Increase by 50%–100%
  C. Increase by 10%–50%
  D. Increase less than 10%
  E. No change
  F. Decrease

*Notes.* In order to show the actual situation in a clear manner, after referring to the opinions of the entrepreneurs, the items 3 and 4 were implemented on a 6-factor subscale.

## Footnotes

1 This abbreviation refers to situations in which digital strategies are immediately successful. SMACIT refers to powerful, easy-to-access digital technology.
